# Clinical N3 is an independent risk factor of recurrence for breast cancer patients achieving pathological complete response and near-pathological complete response after neoadjuvant chemotherapy

**DOI:** 10.3389/fonc.2022.1019925

**Published:** 2022-10-06

**Authors:** Xiaoyan Qian, Meng Xiu, Qing Li, Jiayu Wang, Ying Fan, Yang Luo, Ruigang Cai, Qiao Li, Shanshan Chen, Peng Yuan, Fei Ma, Binghe Xu, Pin Zhang

**Affiliations:** ^1^ Department of Medical Oncology, National Cancer Center/National Clinical Research Center for Cancer/Cancer Hospital, Chinese Academy of Medical Sciences and Peking Union Medical College, Beijing, China; ^2^ Department of Oncology, Henan Provincial People’s Hospital, People’s Hospital of Zhengzhou University, Zhengzhou, China; ^3^ Department of VIP Medical Services, National Cancer Center/National Clinical Research Center for Cancer/Cancer Hospital, Chinese Academy of Medical Sciences and Peking Union Medical College, Beijing, China

**Keywords:** breast cancer, pathological complete response, near-pathological complete response, survival, predictive factors

## Abstract

**Background:**

Although achieving pathological complete response (pCR) and near-pathological complete response (near-pCR) after neoadjuvant chemotherapy (NAC) in breast cancer predicts a better outcome, some patients still experience recurrence. The aim of our study was to investigate the predictive factors of recurrence in the pCR and near-pCR population.

**Methods:**

We reviewed 1,209 breast cancer patients treated with NAC between January 2010 and April 2021 in the Cancer Hospital, Chinese Academy of Medical Sciences (CHCAMS). A total of 292 patients achieving pCR and near-pCR were included in our analysis. pCR was defined as ypT0N0/ypTisN0. Near-pCR was defined as ypT1mi/1a/1bN0 or ypT0/isN1mi. Clinical features and follow-up information were collected. Survival and predictive factors of recurrence were analyzed.

**Results:**

Of the 292 patients, 173 were pCR and 119 were near-pCR. The median age was 46 years (range, 23–75 years). The predominant tumor subtypes were human epidermal growth factor receptor type 2 (HER2)-positive breast cancer (49.0%) and triple-negative breast cancer (TNBC) (30.8%). The median duration of follow-up was 53 months (range, 9–138 months). A total of 25 (8.6%) patients developed recurrence, with 9 (5.2%) in the pCR group and 16 (13.4%) in the near-pCR group. The vast majority of recurrence occurred within 36 months from onset of NAC. The 5-year recurrence-free survival (RFS) rate of patients achieving pCR was significantly higher than that of patients achieving near-pCR (94.6% vs. 85.6%, *p* = 0.008). However, the 5-year overall survival (OS) rate between the two cohorts had no statistical difference (94.3% vs. 89.6%, *p* = 0.304). Clinical N3 (cN3) before NAC was an independent risk factor of recurrence in patients who achieved pCR (*p* = 0.003) and near-pCR (*p* = 0.036). Tumor size before NAC, subtypes of breast cancer, and chemotherapy regimens showed no significant association with RFS both for patients who achieved pCR and for those who achieved near-pCR (*p* > 0.05).

**Conclusions:**

cN3 before NAC was an independent risk factor of recurrence in patients who achieved pCR and near-pCR. It is worthwhile to closely monitor patients with cN3, especially in the first 3 years.

## Introduction

Neoadjuvant chemotherapy (NAC) was widely used in patients with human epidermal growth receptor 2 (HER2)-positive breast cancer and triple-negative breast cancer (TNBC) (1–3). HER2-positive breast cancer and TNBC are relatively sensitive to NAC, and pathological reaction to NAC can provide prognostic information and guide the selection of postoperative treatment (4–9). Due to the rapid development of antineoplastic drugs in recent decades, the rate of pathological complete response (pCR) after NAC has significantly increased (10). Studies have demonstrated that patients achieving pCR had significantly better disease-free survival (DFS) and overall survival (OS) than patients with residual disease (11, 12). The assessment of obtaining a real pCR is of great importance and has been gradually standardized nowadays. The generally accepted definition of pCR is that there is no residual invasive carcinoma in the breast and in all sampled lymph nodes (ypT0/isN0) (13–15). More recently, the concept of near-pCR was gradually being proposed and has attracted more and more attention. Substantial research elucidated that patients who achieved near-pCR also had outstanding DFS and OS (13, 14). A variety of definitions of near-pCR have been used in neoadjuvant clinical trials in breast cancer. The most common consensus was that the residual disease ≤1 cm (9, 16).

In spite of the outstanding outcomes of patients achieving pCR and near-pCR, some of them may still experience recurrence. In order to identify clinical and pathological predictive factors of cancer recurrence, we performed this retrospective analysis among breast cancer patients who achieved pCR and near-pCR in the Cancer Hospital, Chinese Academy of Medical Sciences (CHCAMS). In this study, we aimed to explore the predictive factors associated with recurrence for the patients achieving pCR and near-pCR, and investigate whether the risk for recurrence and death of patients achieving near-pCR was comparable with those achieving pCR.

## Methods

### Study population

We reviewed 1,209 breast cancer patients that were treated with NAC between January 2010 and April 2021 in CHCAMS. The inclusion criteria in this study were as follows: (1) patients who were pathologically diagnosed with invasive breast cancer based on WHO criteria; (2) patients who have early-stage or locally advanced breast cancer (4, 13); (3) patients receiving surgery after NAC; (4) patients with complete clinical information; and (5) patients with follow-up data. Exclusion criteria were as follows: (1) patients with distant metastasis before or during NAC; (2) patients without detailed pathology after surgery; and (3) patients who withdraw active follow-up data. A final cohort of 292 patients who achieved pCR and near-pCR was incorporated in this study. Clinical and pathological data of these patients were collected: age, menstruation, tumor size, regional lymph node, estrogen receptor (ER), progesterone receptor (PR), HER2, Ki67 index, chemotherapy, radiation, endocrine, and surgery regimens.

### Pathological assessment

pCR was defined as no residual invasive carcinoma in the breast and negative axillary lymph nodes, including ypT0N0 and ypTisN0 (13–15). Near-pCR was defined as the residual tumor size ≤1 cm in the breast and negative axillary lymph nodes, or no residual invasive carcinoma in the breast yet existing micrometastasis in lymph node, including ypT1mi/a/bN0 and ypT0/isN1mi (9, 16). Pathologically, T and N were defined according to the AJCC Staging System of Breast Cancer, 8th edition (17).

The Miller–Payne grade system was used to evaluate breast cancer pathological responses to NAC (18). Grade 1: no significant reduction in tumor cells; Grade 2: a minor reduction in tumor cells (≤30%); Grade 3: reduction in tumor cells between 30% and 90%; Grade 4: disappearance of tumor cells > 90%; Grade 5: no invasive tumor cells identifiable, and DCIS may be present.

ER and PR status was assessed by immunohistochemistry (IHC) and categorized as positive when more than 1% of cancer cells were stained (19). HER2 positive was defined as 3+ by IHC or positive by fluorescence *in situ* hybridization (FISH) (20). Ki67 index was defined as the mean tumor cells with marker expression by IHC: low (<20%), intermediate (20%–49%), and high (≥50%) (21–23).

The molecular subtype classification was on the basis of IHC of ER, PR, HER2, and Ki67 (24). Luminal A: ER and PR positive (PR ≥ 20%), HER2 negative, and Ki67 low expression; Luminal B HER2-negative: ER and/or PR positive, HER2 negative; Luminal B HER2-positive: ER and/or PR positive, HER2 positive; HER2-positive (non-luminal): ER and PR negative, HER2 positive; Triple-negative: ER and PR negative, HER2 negative.

Recurrence-free survival (RFS) was calculated as the time from the onset of NAC to local or distant recurrence, or death due to any cause, whichever came first. OS was calculated as the time from the onset of NAC to death due to any cause.

### Statistical analysis

All statistical analyses were conducted using SPSS 25.0 and R (version 3.5.1). The Kaplan–Meier method with the log-rank test was used for recurrence and survival analysis. The factors significant at the 20% level in the univariate analysis were considered for inclusion in the multivariate model. The Cox proportional hazards regression model was used to assess the association of clinical and pathological predictive factors with RFS. C-statistics was conducted to evaluate the predictive value of the factors. All tests were two tailed and a *p*-value less than 0.05 was considered to indicate a statistically significant difference.

## Results

### Patient characteristics

A total of 292 patients with pCR and near-pCR were included in this study. Their clinical and pathological characteristics are described in **Table 1**. The median age of patients was 46 years (range, 23–75 years); 62.3% were premenopausal. The median duration of follow-up for these patients was 53 months (range, 9–138 months). There were 173 patients achieving pCR and 119 achieving near-pCR. The predominant tumor subtypes were HER2 positive (49.0%) (including luminal B HER2+ and non-luminal HER2+) and TNBC (30.8%). Among the patients with HER2 positive, 63.6% received trastuzumab, while 24.4% received trastuzumab and pertuzumab. The majority of the tumors were T2+ (91.4%) and N+ (76.0%). Overall, 77.0% of the patients underwent mastectomy and 83.2% of the patients had axillary lymph node dissection.

**Table 1:** Patient characteristics.

Characteristics	Total (*n* = 292)		pCR (*n* = 173)		Near-pCR (*n* = 119)	
	No.	%	No.	%	No.	%
Median age (range)	46 (23–75)		48 (23–73)		42 (24–75)	
Age						
<40	94	32.2	43	24.9	51	42.9
40–59	168	57.5	110	63.6	58	48.7
≥60	30	10.3	20	11.6	10	8.4
Menopausal status						
Premenopausal	182	62.3	101	58.4	81	68.1
Postmenopausal	110	37.7	72	41.6	38	31.9
cT						
T1	25	8.6	15	8.7	10	8.4
T2	168	57.5	108	62.4	60	50.4
T3	69	23.6	35	20.2	34	28.6
T4	30	10.3	15	8.7	15	12.6
cN						
N0	70	24.0	33	19.1	37	31.1
N1	69	23.6	35	20.2	34	28.6
N2	98	33.6	68	39.3	30	25.2
N3	55	18.8	37	21.4	18	15.1
cTNM						
I	4	1.4	1	0.6	3	2.5
IIA	49	16.8	23	13.3	26	21.8
IIB	51	17.5	29	16.8	22	18.5
IIIA	108	37.0	70	40.5	38	31.9
IIIB	25	8.6	13	7.5	12	10.1
IIIC	55	18.8	37	21.4	18	15.1
ER status						
Negative	203	69.5	128	74.0	75	63.0
Positive	89	30.5	45	26.0	44	37.0
PR status						
Negative	174	59.6	115	66.5	59	49.6
Positive	118	40.4	58	33.5	60	50.4
HER2 status						
Negative	149	51.0	86	49.7	63	52.9
Positive	143	49.0	87	50.3	56	47.1
Ki67						
<20	16	5.5	7	4.0	9	7.6
20-49	117	40.1	65	37.6	52	43.7
≥50	142	48.6	92	53.2	50	42.0
Unknown	17	5.8	9	5.2	8	6.7
Breast cancer subtype						
Luminal A	1	0.3	0	0.0	1	0.8
Luminal B HER2−	58	19.9	30	17.3	29	24.4
Luminal B HER2+	77	26.4	41	23.7	35	29.4
Non-luminal HER2+	66	22.6	45	26.0	21	17.6
Triple negative	90	30.8	57	32.9	33	27.7
Chemotherapy regimens of NAC						
Anthracycline and taxane	86	29.5	40	23.1	46	38.7
Taxane and platinum	175	59.9	117	67.6	58	48.7
Anthracycline and taxane and platinum	15	5.1	10	5.8	5	4.2
Anthracycline or taxane	13	4.5	6	3.5	7	5.9
Endocrine	3	1.0	0	0.0	3	2.5
Cycle number of NAC						
<4	10	3.4	2	1.2	8	6.7
4–6	248	84.9	153	88.4	95	79.8
>6	31	10.6	18	10.4	13	10.9
Other (Endocrine Therapy)	3	1.0	0	0.0	3	2.5
Surgery of breast cancer						
Breast-conserving surgery	67	23.0	41	23.7	26	21.8
Mastectomy	225	77.0	132	76.3	93	78.2
Surgery of lymph nodes						
Sentinel lymph node biopsies	49	16.8	28	16.2	21	17.6
Axillary lymph node dissection	243	83.2	145	83.8	98	82.4
Adjuvant radiation						
Yes	211	72.3	125	72.3	86	72.3
No	81	27.7	48	27.7	33	27.7
Adjuvant endocrine						
Yes	128	43.8	67	38.7	61	51.3
No	164	56.2	106	61.3	58	48.7
HER2 positive						
With trastuzumab	91	63.6	59	67.8	32	57.1
With trastuzumab and pertuzumab	35	24.4	23	26.4	12	21.4
With trastuzumab and TKI	1	0.7	0	0	1	1.8
Without HER2 targeted therapy	16	11.1	5	5.7	11	19.6

pCR, pathological complete response; Near-pCR, near-pathological complete response; cT, clinical tumor size; cN, clinical lymph node status; ER, estrogen receptor; PR, progesterone receptor; HER2, human epidermal growth factor receptor type 2; Ki67, Ki67 index; NAC, neoadjuvant chemotherapy; TKI, tyrosine kinase inhibitor.

### Disease recurrence

As shown in **Table 2**, a total of 25 (8.6%) patients developed recurrence. Twenty-one (84.0%) recurrences occurred within 36 months. Among patients achieving pCR, 9 (5.2%) patients developed cancer recurrence, with 2 patients presenting with both local recurrence and distant metastasis, while 7 patients presented with distant metastasis. The median time to recurrence was 14 months (range, 8–62 months) from the onset of NAC. Four (44.4%) patients presented liver metastasis and 2 (22.2%) patients presented brain metastasis as the first event.

**Table 2:** Time and site of recurrence.

	pCR		Near-pCR	
	*N*	%	*N*	%
	9		16	
Median (range), months	14 (8–62)		18 (4–69)	
≤12 months	3	33.3	5	31.3
12–36 months	4	44.4	9	56.3
>36 months	2	22.2	2	12.5
Site of disease recurrence				
Local recurrence	2	22.2	8	50.0
Breast or chest wall	1	11.1	3	18.8
Regional lymph nodes	1	11.1	5	31.3
Distant metastasis	9	100	12	75.0
Liver	4	44.4	1	6.3
Lung	1	11.1	3	18.8
Brain	2	22.2	2	12.5
Bone	0	0	6	37.5
Other sites	2	22.2	2	12.5

pCR, pathological complete response; Near-pCR, near-pathological complete response.

With regard to patients achieving near-pCR, 16 (13.4%) patients developed cancer recurrence, with 4 patients presenting with local recurrence only, 4 patients with both local recurrence and distant metastasis, while 8 patients presenting with distant metastasis only. The median time to recurrence was 18 months (range, 4–69 months). Three (18.8%) patients experienced lung metastasis and 6 (37.5%) patients presented bone metastasis as the first event.

### RFS and OS

**Figure 1:**
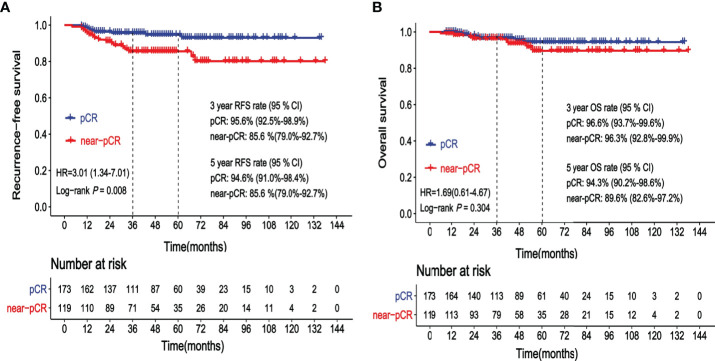
**(A)** Kaplan–Meier curve showing recurrence-free survival (RFS) according to the status after neoadjuvant chemotherapy (NAC): pathological complete response (pCR) vs. near-pathological complete response (near-pCR). **(B)** Kaplan–Meier curve showing overall survival (OS) according to the status after neoadjuvant chemotherapy (NAC): pathological complete response (pCR) vs. near-pathological complete response (near-pCR).

The 3-year RFS rates of patients achieving pCR and near-pCR were 95.6% and 85.6%, respectively. The 5-year RFS rates of patients achieving pCR and near-pCR were 94.6% and 85.6%, respectively. The risk of cancer recurrence was significantly higher in patients achieving near-pCR than that in patients achieving pCR (HR = 3.01, 95% CI: 1.34–7.01, *p* = 0.008, **Figure 1A**). A total of 15 (5.1%) patients died. The 3-year OS rates of the pCR group and the near-pCR group were 96.6% and 96.3%, respectively. The 5-year OS rates of the pCR and near-pCR groups were 94.3% and 89.6%, respectively. There was no statistical difference in OS between the two cohorts (HR = 1.69, 95% CI: 0.61–4.67, *p* = 0.304, **Figure 1B**).

### Predictive factors of RFS in patients achieving pCR

**Figure 2:**
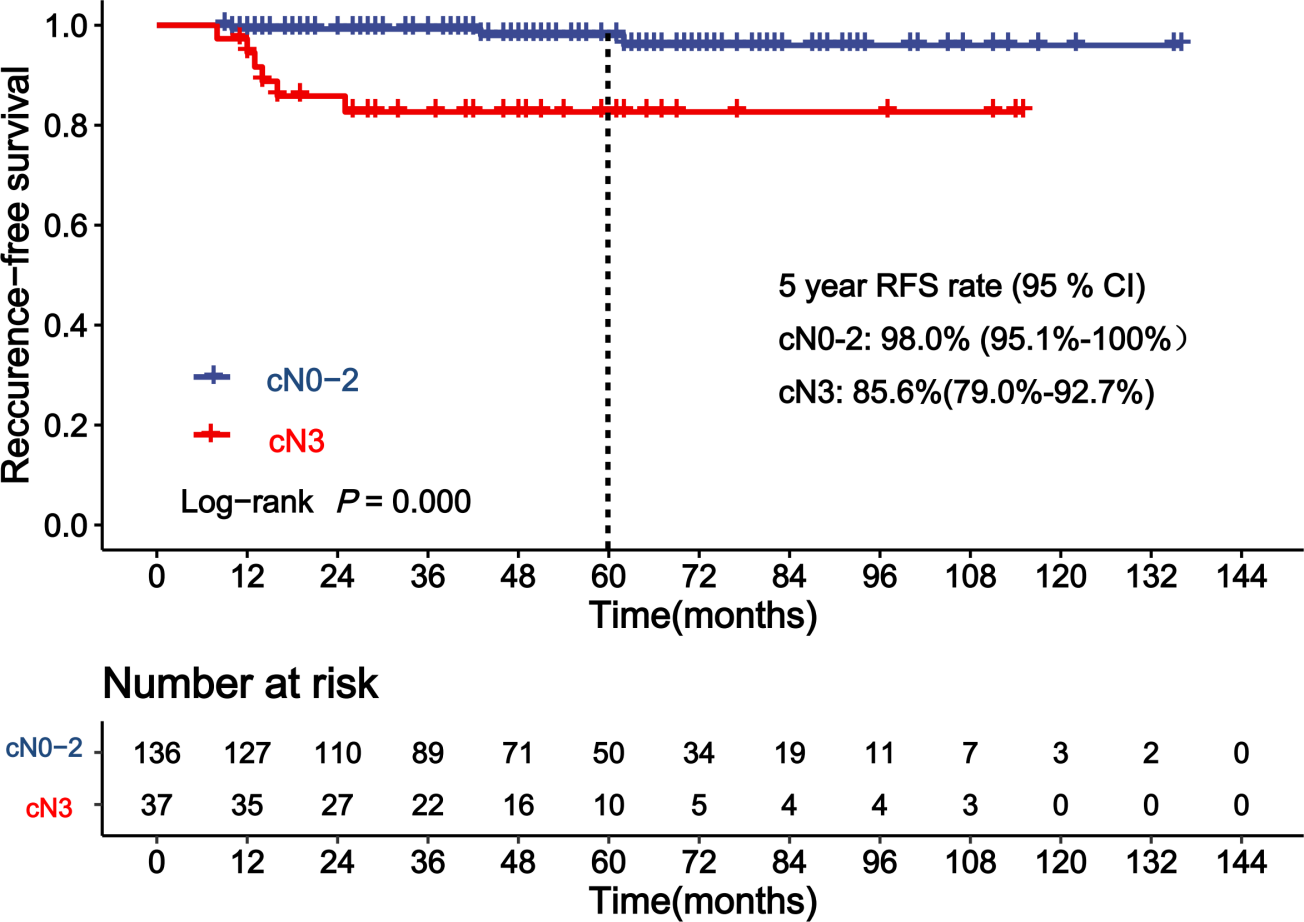
Kaplan–Meier curve showing recurrence-free survival (RFS) of patients achieving pathological complete response (pCR) according to clinical lymph node status (cN).

**Table 3** shows the results of the analyses for factors associated with RFS of patients achieving pCR. Clinical lymph node status (cN) before NAC was a significant covariate in the univariate analysis for RFS in patients achieving pCR (*p* < 0.001). The 5-year RFS rates for cN0–2 and cN3 patients who achieved pCR were 98.0% and 82.7%, respectively. cN3 was an independent factor of higher risk for recurrence on the multivariate analysis (**Figure 2**, HR = 9.8, 95% CI: 2.1–44.5, *p* = 0.003). The C-statistics was 0.77 (95% CI: 0.63–0.91) of cN3 for RFS prediction. Age at diagnosis, tumor size at diagnosis, subtypes of breast cancer, and other factors showed no significant association with RFS of patients who achieved pCR (*p >* 0.05).

**Table 3:** Analysis of predictive factors for RFS in patients who achieved pCR.

	Univariate analysis				Multivariate analysis		
	*N*	Events	5-year RFS rate (%) (95% CI)	*P-*value	HR (95% CI)	*P*-value	C-statistics (95% CI)
Total	173	9					
Age				0.416			
<40	44	3	92.0 (83.7–100)				
≥40	129	6	95.5 (88.2–99.1)				
Menopausal status				0.919			
Premenopausal	101	5	95.8 (91.9–99.9)				
Postmenopausal	72	4	93.3 (87.0–100)				
cT				0.730			
T1–2	123	5	96.3 (92.8–99.9)				
T3–4	50	4	91.1 (83.0–100)				
cN				0.000			
N0–2	136	3	98.0 (95.1–100)		Reference		
N3	37	6	82.7 (70.9–96.3)		9.8 (2.1–44.5)	**0.003**	**0.77** (**0.63–0.91**)
ER status				0.154			
Negative	128	5	96.2 (92.5–100)		Reference		
Positive	45	4	89.7 (80.5–99.9)		0.9 (0.2–4.2)	0.939	
PR status				0.151			
Negative	115	4	96.6 (92.8–100)		Reference		
Positive	58	5	90.7 (83.2–98.8)		2.2 (0.5–9.1)	0.296	
HER2 status				0.737			
Negative	86	4	95.0 (90.3–99.9)				
Positive	87	5	94.2 (88.6–100)				
Ki67				0.623			
<50	72	5	93.0 (86.5–100)				
≥50	92	4	95.5 (91.2–99.9)				
unknown	9	0	—				
Breast cancer subtype				0.750			
Luminal*	29	2	92.1 (82.3–100)				
HER2 positive**	87	5	94.2 (88.6–100)				
Triple negative	57	2	96.4 (91.6–100)				
Chemotherapy regimens of NAC				0.063			
Anthracycline and taxane	40	5	87.4 (77.7–98.4)		Reference		
Taxane and platinum	117	3	98.2 (95.9–100)		0.3 (0.1–1.4)	0.116	
Others	16	1	85.7 (63.3–100)		2.1 (0.2–22.7)	0.534	
Cycle number of NAC				0.591			
<4	2	0	—				
4–6	153	9	94.0 (90.0–98.2)				
>6	18	0	—				
Surgery of breast cancer				0.359			
Breast-conserving surgery	41	3	94.5 (87.4–100)				
Mastectomy	132	6	94.8 (90.7–99.0)				
Surgery of lymph nodes				0.873			
Sentinel lymph node biopsies	28	1	100				
Axillary lymph node dissection	145	8	93.8 (89.6–98.1)				
Adjuvant radiation				0.695			
Yes	125	7	94.2 (89.8–98.9)				
No	48	2	95.5 (89.5–100)				
Adjuvant endocrine				0.234			
Yes	67	5	91.5 (84.6–99.0)				
No	106	4	96.5 (92.5–100)				

RFS, recurrence-free survival; pCR, pathological complete response; cT, clinical tumor size; cN, clinical lymph node status; ER, estrogen receptor; PR, progesterone receptor; HER2, human epidermal growth factor receptor type 2; Ki67, Ki67 index; NAC, neoadjuvant chemotherapy.

*Luminal included luminal A and luminal B HER2-.

**HER2 positive included luminal B HER2+ and non-luminal HER2+.

The bold value means having statistical difference.

### Predictive factors of RFS in patients achieving near-pCR

**Figure 3:**
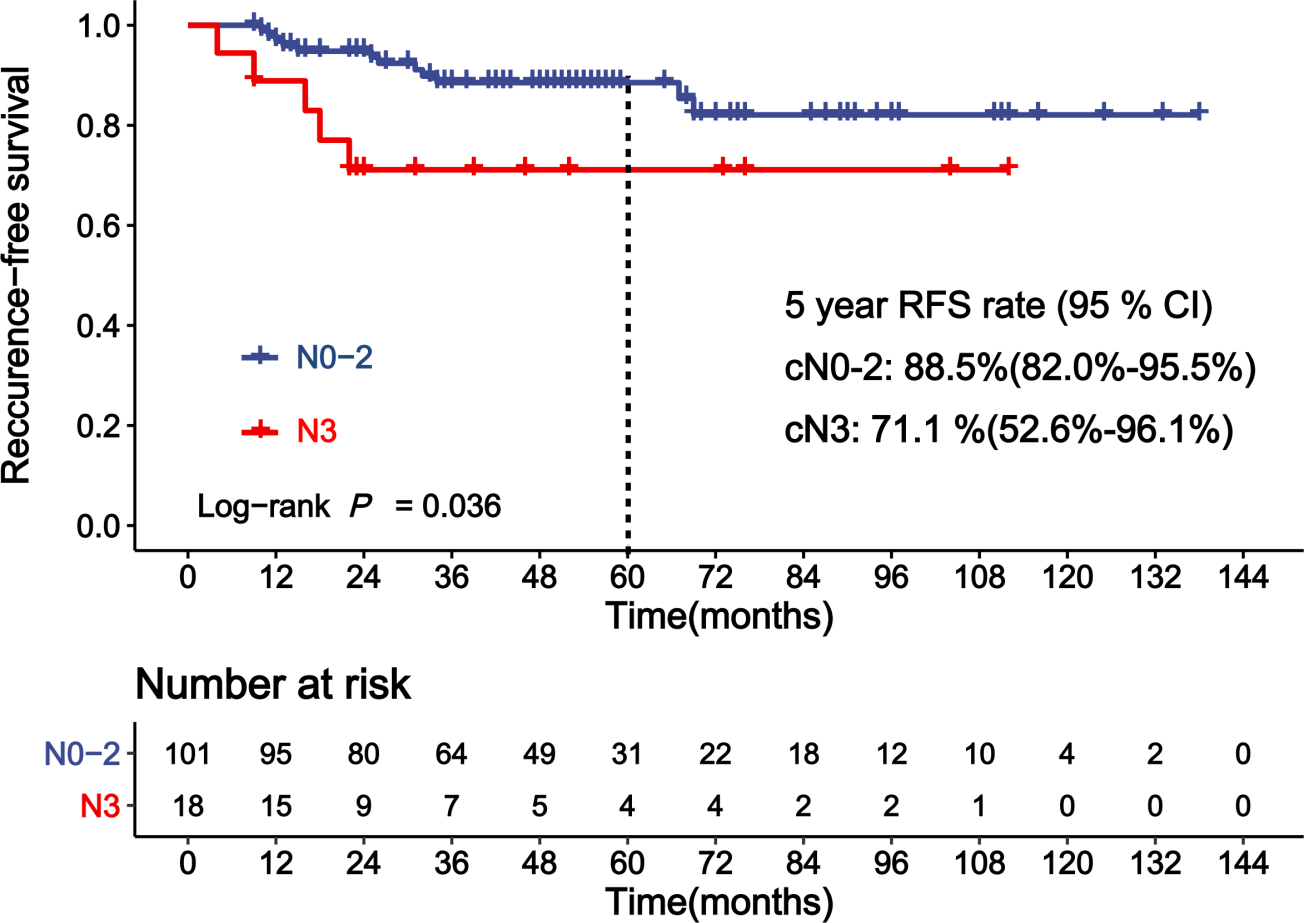
Kaplan–Meier curve showing recurrence-free survival (RFS) of patients achieving near-pCR according to clinical lymph node status (cN).

**Table 4** shows the results of the analyses for the factors associated with RFS of patients achieving near-pCR. cN before NAC was a significant covariate in the univariate analysis for RFS in patients achieving near-pCR (**Figure 3**, *p* = 0.036). The 5-year RFS rates for cN0–2 and cN3 patients who achieved near-pCR were 88.5% and 71.1%, respectively. The C-statistics was 0.63 (95% CI: 0.52–0.74) of cN3 for RFS prediction. There was no difference between ypT1miN0, ypT1aN0, and ypT1bN0 for RFS (*p* = 0.942). The Miller–Payne grade after NAC also showed no significant association with the RFS of patients who achieved near-pCR (*p >* 0.05). There were no other factors significant at the 20% level in the univariate analyses of RFS for patients achieving near-pCR; thus, we did not conduct multivariate analyses further.

**Table 4:** Analyses of predictive factors for RFS in patients who achieved near-pCR.

	Univariate analyses				
	*N*	Events	5-year RFS rate (%) (95% CI)	*P-*value	C-statistics (95% CI)
Total	119	17			
Age				0.251	
<40	51	9	81.5 (70.6–94.1)		
≥40	68	8	88.5 (80.8–97.0)		
Menopausal status				0.467	
Premenopausal	81	13	84.7 (76.7–93.5)		
Postmenopausal	38	4	87.6 (76.7–100)		
cT				0.506	
T1–2	70	12	83.6 (74.6–93.6)		
T3–4	49	5	88.5 (79.4–98.6)		
cN				**0.036**	**0.63** (**0.52–0.74**)
N0–2	101	12	88.5 (82.0–95.5)		
N3	18	5	71.1 (52.6–96.1)		
ER status				0.720	
Negative	75	10	86.5 (78.6–95.2)		
Positive	44	7	83.8 (72.7–96.7)		
PR status				0.411	
Negative	59	10	83.4 (74.1–94.0)		
Positive	60	7	87.2 (78.2–97.4)		
HER2 status				0.300	
Negative	56	6	90.8 (82.5–99.9)		
Positive	63	11	80.9 (71.3–91.8)		
Ki67				0.740	
<50	61	10	83.1 (73.5–93.9)		
≥50	50	6	85.9 (76.0–97.2)		
Unknown	8	1	—		
Breast cancer subtype				0.583	
Luminal*	30	5	80.3 (66.2–97.5)		
HER2 positive**	56	6	90.8 (82.5–99.9)		
Triple negative	33	6	81.4 (68.9–96.0)		
Treatment of NAC				0.476	
Anthracycline and taxane	46	5	90.5 (82.0–99.8)		
Taxane and platinum	58	10	83.0 (73.4–93.9)		
Others	15	2	77.1 (53.5–100)		
Cycle number of NAC				0.944	
<4	8	1	75.0 (42.6–100)		
4–6	95	14	86.0 (79.0–93.8)		
>6	13	2	81.5 (61.1–100)		
Surgery of breast cancer				0.610	
Breast-conserving surgery	26	3	87.8 (75.8–100)		
Mastectomy	93	14	84.6 (76.8–93.1)		
Surgery of lymph nodes				0.432	
Sentinel lymph node biopsies	21	4	76.3 (58.0–100)		
Axillary lymph node dissection	98	13	87.3 (80.5–94.7)		
ypTNM after NAC				0.942	
ypT1miN0M0	5	1	80.0 (51.6–100)		
ypT1aN0M0	73	11	86.1 (78.0–95.0)		
ypT1bN0M0	39	5	85.0 (73.5–98.2)		
ypT0N1miM0	2	0	—		
Miller–Payne grade				0.334	
1–3	42	4	89.2 (79.8–99.8)		
4–5^#^	77	13	83.8 (75.5–93.1)		
Adjuvant radiation				0.545	
Yes	86	12	86.1 (78.7–94.1)		
No	33	5	83.8 (70.4–99.8)		
Adjuvant endocrine				0.925	
Yes	61	9	84.1 (74.5–95.9)		
No	58	8	86.9 (78.2–96.5)		

RFS, recurrence-free survival; near-pCR, near-pathological complete response; cT, clinical tumor size; cN, clinical lymph node status; ER, estrogen receptor; PR, progesterone receptor; HER2, human epidermal growth factor receptor type 2; Ki67, Ki67 index; NAC, neoadjuvant chemotherapy.

*Luminal included luminal A and luminal B HER2-.

**HER2 positive included luminal B HER2+ and non-luminal HER2+.

^#^Two patients with Miller–Payne grade 5 were ypT0N1miM0.

The bold value means having statistical difference.

## Discussion

In this retrospective study of 292 patients achieving pCR and near-pCR after NAC, the recurrence pattern of patients was described, and the vast majority of recurrence occurred within 36 months from onset of NAC. This study found that the risk for recurrence of patients achieving near-pCR after NAC was higher than those achieving pCR. Moreover, cN3 before NAC was identified as a robust predictive factor of RFS for patients achieving pCR and near-pCR.

The 5-year RFS rate of pCR was 94.6% in our study, which was consistent with previous studies (25–27). The sub-study of EORTC 10994/BIG 1-00 phase III trial including 283 patients found that clinical tumor size was the only predictor for distant recurrence-free interval (DRFI) after pCR (27). In the research from the Anderson group, the authors identified that clinical stage IIIB–C and inflammatory breast cancer, premenopausal status, and resection of fewer than 10 lymph nodes were associated with an increased risk of developing distant metastasis for patients achieving pCR (28). Since cN contributes to the clinical stage, our study was partly consistent with the Anderson research. The predictive value of cN3 was also confirmed by C-statistics (0.77, 95% CI: 0.63–0.91) and Cox proportional hazards regression (cN3 vs. cN0–2, HR = 9.8, 95% CI: 2.1–44.5, *p* = 0.003). Asaoka and colleagues’ research also found that lymph node metastasis before NAC was the only predictor of cancer recurrence on multivariate analyses for patients achieving pCR (29).

The 5-year RFS rate of near-pCR was 85.6%, which was 9% lower than that of patients achieving pCR, but the OS of the two cohorts had no significant difference. The Spring et al. meta-analysis (30), which included 27,895 patients from 52 publications, showed that patients with residual disease after NAC had a 5-year DFS rate of 67%, which was much lower compared with the near-pCR population (85.6%) in our study. This illustrated the fact that it was necessary to distinguish the near-pCR population from the residual disease. There has been controversy regarding the definition of near-pCR in the past few years (31). In Cheng’s study, near-pCR was defined as residual tumor volume <1 cm^3^ (16). While residual tumor size ≤ 1 cm was excluded in KATHERINE, a clinical trial focused on intensive postoperative treatment (9). However, Lee and colleagues defined near-pCR as tumor size ≤ 0.5 cm (32). In our study, near-pCR was defined as the residual tumor size ≤1 cm in the breast (ypT1mi/1a/1bN0), or no residual invasive carcinoma in the breast yet existing micrometastasis in lymph node (ypT0/isN1mi).

To our best knowledge, this study is the first one to report the potential predictive factors of RFS for patients achieving near-pCR. We found that cN3 was an independent factor of higher risk for recurrence in the near-pCR subgroup, which was consistent with the pCR subgroup. The 5-year RFS rates for cN0–2 and cN3 patients who achieved near-pCR were 88.5% and 71.1%, respectively (*p* = 0.036). According to AJCC 8th edition staging system of breast cancer, cN3 is defined as metastasis to ipsilateral infraclavicular/supraclavicular lymph node(s), or metastasis to ipsilateral internal mammary lymph node(s) and axillary lymph node(s). There is controversy regarding the treatment of the local supraclavicular and internal mammary lymph node(s). It is difficult to remove the supraclavicular lymph node(s) and internal mammary lymph node(s) during the surgery. Radiation therapy is usually applied to deal with the supraclavicular and internal mammary lymph node(s) involvement. However, it is difficult to evaluate whether the status of no evidence of disease (NED) is reached. In recent years, growing interest was focused on post-NAC treatment, and some trials noted that reinforcing the adjuvant treatment could improve prognosis for patients with residual disease. In the subset of CREATE-X, TNBC patients with residual invasive disease who received capecitabine had a 5-year DFS rate of 69.8%, 13.7% higher than the control group (HR = 0.58, 95% CI: 0.39–0.87) (8). In the KATHERINE clinical trial, the invasive DFS at 3 years of HER2-positive breast cancer patients with residual invasive disease who received T-DM1 was 88.3%, higher than patients receiving trastuzumab (HR = 0.5, *p* < 0.001) (9). However, numerous post-NAC clinical trials incorporated patients with a residual disease of at least 1.0 cm or node positive disease, excluding patients who achieved near-pCR. Our study showed that patients with near-pCR still had a certain risk of recurrence. Adjuvant therapy to minimize the risk of recurrence for patients with near-pCR is needed to be illuminated in further prospective research.

Our study also has several limitations. First, it was a retrospective study; therefore, selection bias was inevitable. Second, because the number of death events was small, we did not conduct analysis on the predictive factors of OS in patients achieving pCR and near-pCR.

## Conclusions

Patients achieving pCR had excellent outcomes. The recurrence risk of patients achieving near-pCR after NAC was higher than that of patients achieving pCR. The vast majority of recurrence occurred within 3 years from onset of NAC. Patients with cN3 before NAC had a higher risk of developing local and distant metastasis, even achieving pCR or near-pCR after NAC. It is worthwhile to closely monitor patients with cN3, especially in the first 3 years.

## Data availability statement

The raw data supporting the conclusions of this article will be made available by the authors, without undue reservation.

## Ethics statement

The studies involving human participants were reviewed and approved by the National Cancer Center/National Clinical Research Center for Cancer/Cancer Hospital, Chinese Academy of Medical Sciences and Peking Union Medical College. Written informed consent for participation was not required for this study in accordance with the national legislation and the institutional requirements.

## Author contributions

PZ contributed to the study concept, design, and patient management, and revised the manuscript. XQ contributed to data collection and data analysis, and drafted the manuscript. MX revised the manuscript. QinL, JW, YF, YL, RC, QiaL, SC, PY, FM, BX participated in patient management. All authors approved the final version of the manuscript.

## Funding

This project was funded by Cancer Prevention and Research Fund of China Medical Foundation and the fund supported the follow-up and publication costs.

## Acknowledgments

The authors wish to thank all the study participants and research staff who participated in this work.

## Conflict of interest

The authors declare that the research was conducted in the absence of any commercial or financial relationships that could be construed as a potential conflict of interest.

## Publisher’s note

All claims expressed in this article are solely those of the authors and do not necessarily represent those of their affiliated organizations, or those of the publisher, the editors and the reviewers. Any product that may be evaluated in this article, or claim that may be made by its manufacturer, is not guaranteed or endorsed by the publisher.
